# Multiplex Networks for Early Diagnosis of Alzheimer's Disease

**DOI:** 10.3389/fnagi.2018.00365

**Published:** 2018-11-14

**Authors:** Nicola Amoroso, Marianna La Rocca, Stefania Bruno, Tommaso Maggipinto, Alfonso Monaco, Roberto Bellotti, Sabina Tangaro

**Affiliations:** ^1^Dipartimento Interateneo di Fisica “M. Merlin”, Università degli studi di Bari “A. Moro”, Bari, Italy; ^2^Dipartimento Interateneo di Fisica “M. Merlin”, Istituto Nazionale di Fisica Nucleare, Sezione di Bari, Bari, Italy; ^3^Laboratory of Neuro Imaging, USC Stevens Neuroimaging and Informatics Institute, Keck School of Medicine of USC, University of Southern California, Los Angeles, CA, United States; ^4^Blackheath Brain Injury Rehabilitation Centre, London, United Kingdom

**Keywords:** multiplex networks, machine learning, diagnosis support system, Alzheimer's disease, mild cognitive impairment, magnetic resonance imaging (MRI), brain Connectivity

## Abstract

Analysis and quantification of brain structural changes, using Magnetic Resonance Imaging (MRI), are increasingly used to define novel biomarkers of brain pathologies, such as Alzheimer's disease (AD). Several studies have suggested that brain topological organization can reveal early signs of AD. Here, we propose a novel brain model which captures both intra- and inter-subject information within a multiplex network approach. This model localizes brain atrophy effects and summarizes them with a diagnostic score. On an independent test set, our multiplex-based score segregates (i) normal controls (NC) from AD patients with a 0.86±0.01 accuracy and (ii) NC from mild cognitive impairment (MCI) subjects that will convert to AD (cMCI) with an accuracy of 0.84±0.01. The model shows that illness effects are maximally detected by parceling the brain in equal volumes of 3, 000 mm^3^ (“patches”), without any *a priori* segmentation based on anatomical features. The multiplex approach shows great sensitivity in detecting anomalous changes in the brain; the robustness of the obtained results is assessed using both voxel-based morphometry and FreeSurfer morphological features. Because of its generality this method can provide a reliable tool for clinical trials and a disease signature of many neurodegenerative pathologies.

## 1. Introduction

Alzheimer's disease (AD) is a progressive, neurodegenerative disease accounting for most cases of dementia after the age of 65. It is expected that over 115 million people will develop AD by 2050 (Alzheimer's Association, 2018). Illness related brain changes can be detected *in vivo* with Magnetic Resonance Imaging (MRI) and neuroimaging has been playing an increasingly important role for the diagnosis of neurodegenerative disorders (Bron et al., 2015; Wei et al., 2016; Lebedeva et al., 2017) to the extent that it has been incorporated in the diagnostic criteria for AD (McKhann et al., 2011). It is now accepted that the neurodegenerative cascade in AD begins in the brain years, decades even, before the clinical and radiological manifestations of the illness. The dementia is preceded by a prodromal phase of mild cognitive impairment (Albert et al., 2011), and this, in turn, by a pre-clinical phase (Sperling et al., 2011) of variable duration. Understanding the biological changes, occurring in these early phases, is of paramount importance, as it would open a window of opportunity for future disease-modifying treatments. While it is clear that neurodegeneration in AD occurs in a rather stereotyped fashion in the majority of cases (West et al., 1994; Perl, 2010; Landin-Romero et al., 2017), it is not known exactly what drives the propagation of the disease within an individual, and what is behind the variations in the patterns of atrophy between individuals. To which extent neurodegeneration propagates through anatomical contiguity is yet to be clarified.

MRI can provide significant information on topological organization of the brain (Yao et al., 2010; Bullmore and Bassett, 2011; Alexander-Bloch et al., 2012; Tijms et al., 2013b), thus graph theory has been widely used to study AD which is known to involve both a structural and a functional disruption of brain connectivity (He et al., 2008; Stam et al., 2009; Ciftçi, 2011; de Haan et al., 2012). These studies reported altered local and global graph properties, supporting the clinical relevance of brain networks, especially within group-wise association studies (Crossley et al., 2014; Daianu et al., 2015).

Up to now, graph models of the brain have been based on two distinct approaches (Suk et al., 2014): (i) voxel-wise and (ii) region of interest analyses. We propose here a novel approach based on parceling MRI brain scans in rectangular boxes, that we call “patches,” of fixed dimensions representing the nodes of a network. Then, we measure pairwise similarity measurements between the nodes to define network connections. Therefore, our approach does not inherit the intrinsic computational burden and lack of statistical power affecting voxel wise descriptions (Davatzikos, 2004). Besides, as it is based on unsupervised segmentations of the brain, it avoids *a priori* assumptions about localization of disease effects and typical bias deriving from segmentation errors (Amoroso et al., 2015). In addition, as brain disease has often a diffuse effect, affecting multiple voxels, but not necessarily corresponding to entire anatomical structures, the proposed approach has the potential to better suit the description of pathological changes in the brain, reflecting biological variability.

Specifically for network science, recent studies have investigated the limitations of traditional approaches to describe real systems (Mucha et al., 2010; Lee et al., 2012; Boccaletti et al., 2014) and have pointed out that context information plays a fundamental role. Analogously, we introduce here the novel perspective of multiplex networks (from now onward also multiplexes). Multiplexes are multi-layer systems with a fixed number of nodes that can be linked in different interacting layers, to investigate inter-subject characterization, rather than group-wise differences. In this study, multiplex-based measures are investigated to detect subtle brain atrophy effects, taking into account inter-subject variability; then, proper measures are used to feed random forest classifiers and reveal the emergence of statistically significant AD-related patterns altering the topological organization of the brain.

## 2. Materials and methods

### 2.1. Subjects

In this study we used a training set $D_{train}$ composed of 67 T1 MRI scans. The sample, described in Boccardi et al. (2015), includes 29 normal controls (NC) and 38 AD subjects from the Alzheimer's Disease Neuroimaging Initiative (ADNI). We also employed an independent test set of 148 subjects $D_{test}$, composed by 52 NC, 48 AD and 48 subjects with mild cognitive impairment converting to AD (cMCI). Conversions to AD occurred in a range of [30, 108] months following the baseline diagnosis. $D_{test}$ subjects were randomly chosen within the whole ADNI in order to match the demographic characteristics of training subjects. The training sample (67) and the test sample (148) are of sufficient size for the construction of robust classification models (Mukherjee et al., 2003; Beleites et al., 2013). All 215 participants underwent whole-brain MRI at 34 different sites. Both 1.5 and 3.0 T scans were included in $D_{train}$ and $D_{test}$. Indeed, 1.5 and 3 T scans do not significantly differ in their power to detect neurodegenerative changes as shown in Ho et al. (2010)

ADNI images consisted of MPRAGE MRI brain scans, which were normalized with the MNI152 brain template of size of 197 × 233 × 189 mm^3^ and resolution of 1 × 1 × 1 mm^3^; as a consequence in the following paragraphs voxels and mm^3^ will be interchangeably used. Clinical and demographic information, including the Mini Mental State Examination (MMSE) score, age, years of education and gender for the $D_{train}$ and $D_{test}$ is detailed in Table 1. Except for MMSE scores, there were no significant differences among the three groups.

**Table 1:** Group size and gender information are reported for each class.

	**$D_{train}$**		**$D_{test}$**			**Total**
Disease status	AD (38)	NC (29)	AD (48)	NC (52)	cMCI (48)	215
Female/male	18/20	13/16	22/26	25/27	21/27	99/116
Age (years)	73.55 ± 8.00	74.97 ± 6.30	78.41 ± 6.02	74.77 ± 6.01	76.12 ± 5.89	75.56 ± 6.44
Education (years)	15.29 ± 3.43	16.93 ± 2.78	15.42 ± 3.13	16.37 ± 2.98	15.33 ± 3.03	15.87 ± 3.07
MMSE	22.68 ± 2.27	29.07 ± 0.92	24.32 ± 1.89	28.97 ± 0.81	26.78 ± 1.77	26.36 ± 1.53
n sites	23	19	26	29	18	34

*The table also provides age, years of education and MMSE (mean and standard deviation). The disease status reported is referred at the baseline visit. According to t-test statistics, MMSE scores resulted significantly different with a p-value p < 0.01 for all comparisons except those between $D_{train}$ and $D_{test}$ normal controls and between $D_{train}$ and $D_{test}$ AD patients*.

**Figure 1:**
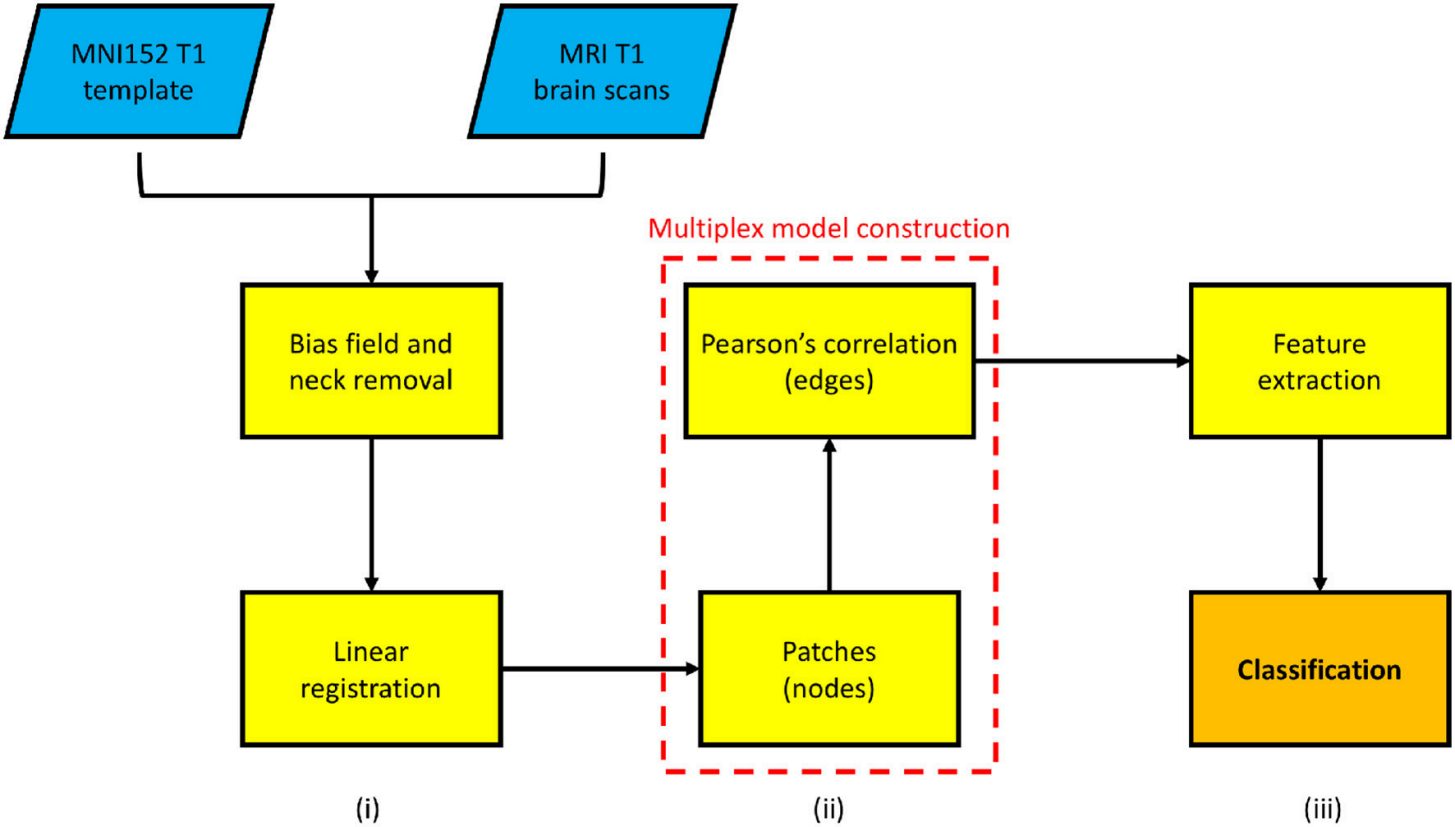
A schematic overview of the proposed framework is presented. In particular: (i) an image pre-processing phase, consisting of intensity and spatial normalization, is necessary to acquire a rough inter-subject correspondence; (ii) then each subject is employed to build a multiplex network (in the dotted box); (iii) finally, machine learning classification is used to assess the multiplex feature information content.

The study encompassed three principal phases: image processing, multiplex network analysis and information content assessment. The first phase is devoted to data normalization, it consists of processing steps which mitigate data heterogeneity; secondly, a network model is assigned to each subject and the comprehensive multiplex model describing the whole cohort is built; finally, quantitative measures are extracted from the model and are used to train a classifier. The overall processing pipeline is schematically represented in Figure 1 and will be explained in detail in the following sections.

### 2.2. Image processing

The nodes of the networks describing each subject should share the same anatomical content in order to be compared. Thus, the proposed approach requires that the same anatomical regions should roughly overlap in order to be robust to subtle local differences, due for example to subject morphological variability, or small registration failures.

Accordingly, intra-cranial regions were extracted and MRI scan intensity differences, yielded by bias field, were normalized with the Oxford FMRIB library FSL (Jenkinson et al., 2012). Then, spatial normalization was performed to co-register the different images into the common coordinate space provided by the MNI152 template. An affine registration was performed with the FSL Linear Registration Tool (FLIRT) with a standard parameter configuration.

**Figure 2:**
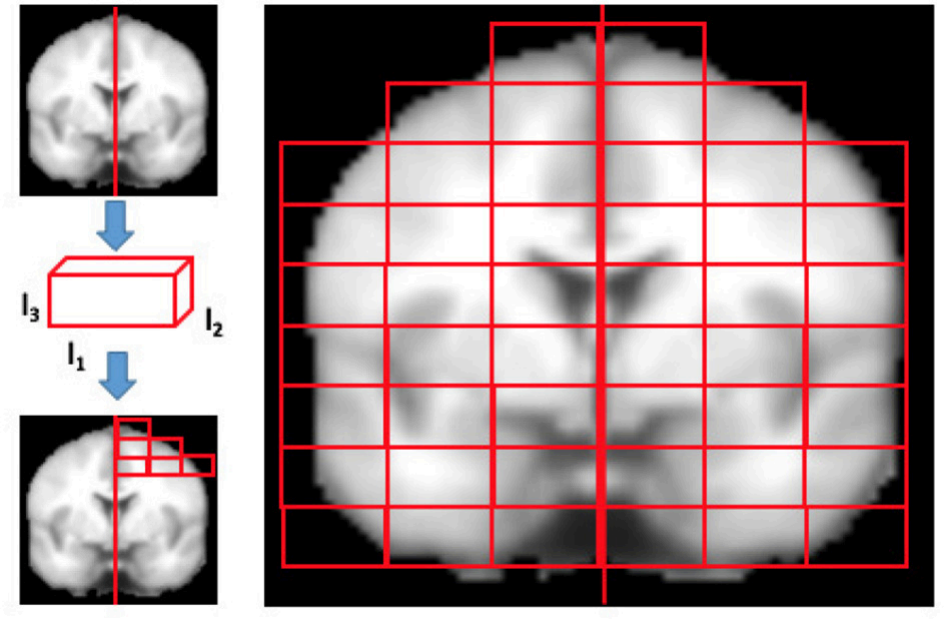
The figure qualitatively shows how MRI brain scans are segmented in rectangular patches of dimensions *l*_1_×*l*_2_×*l*_3_. Firstly, the brains normalized to MNI152 template are divided in left and right hemispheres using the medial longitudinal fissure, then the patch dimensions are set and finally the brain is segmented. Only patches overlapping the brain for at least the 10% of their content are kept, others are discarded.

Finally, we divided the brain of each subject into the two hemispheres by the medial longitudinal fissure. Starting from this sagittal plane, it was possible to uniformly cover each hemisphere with an equal number of rectangular (*l*_1_×*l*_2_×*l*_3_) boxes, from now onward referred to as “patches,” covering the whole brain, see Figure 2. It is worth noting that, once the MRI scans and the template had been co-registered, they shared the same reference space and therefore the anatomical content of each patch was almost the same.

The size *D* of the patches was chosen considering that too small patches could be considerably affected by registration noise, while a size too large, may make it impossible to distinguish subtle disease effects, often diffused to different parts of a region, due to natural inter-subject variability. To investigate how the size of patches affected the quality of the analysis, the overall patch volume *D* was varied from a minimum of 1, 000 to a maximum of 4, 000 voxels. The *l*_1_, *l*_2_, and *l*_3_ values were chosen in order to obtain patches whose dimensions were divisor of the image size and divided regularly the image. Then, only the patches whose voxels overlapped the template brain mask more than 10% were considered.

The patches were considered nodes of a network whose connections represented the grade of similarity between them. We, therefore, used different similarity metrics and a multiplex network framework, in order to extract inter- and intra-subject characteristics.

### 2.3. Multiplex network construction

**Figure 3:**
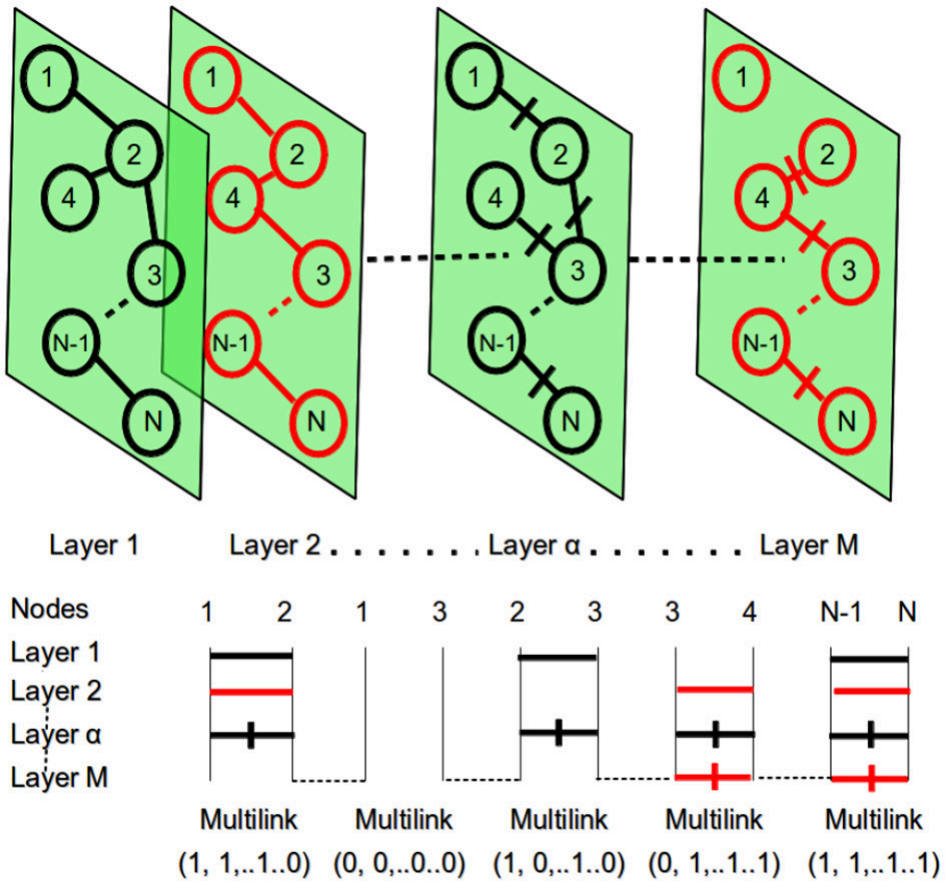
At the top: the multiplex network with *M* layers and *N* nodes. At the bottom: the representation of multi-links for the different pairs of network nodes. Within each layer different nodes can be connected with a link and a specific weight. This context information is then used to detect different patterns.

Graph theory provides tools to concisely quantify the properties of complex networks that describe interrelationships (represented by edges) between the objects of interest (represented by nodes). In this work, for each image and, thus, for each subject, we built an *N* node undirected weighted network with nodes defined by brain MRI patches and edges defined by pairwise Pearson's correlation among them. Therefore, multiplex network $G=\left\{G_{1},G_{2},\ldots ,G_{\alpha },\ldots ,G_{M}\right\}$ was, in this case, a collection of single subject weighted networks $G_{\alpha }=\left(N,E_{\alpha },W_{\alpha }\right)$ (see Figure 3 for a pictorial representation) sharing a common number of nodes *N*, while the set of links $E_{\alpha }$ changed depending on the layer (subject) α. Each network $G_{\alpha }$ can also be represented by the corresponding adjacency matrix $A_{\alpha }=a_{ij}^{\alpha }$, a useful notation to investigate the network properties.

Hence, the proposed model is a multiplex composed of *M* = 67 weighted undirected networks: each representing an MRI brain scan, and including *N* nodes or patches. For each layer, interrelationships were described by $W_{\alpha }=\left\{w_{ij}^{\alpha }\right\}$ in which *w*_*ij*_ were given in terms of Pearson's correlation. In particular, given patches *s*_*i*_ and *s*_*j*_ of dimension *D*, the Pearson's correlation coefficient *r*_*ij*_ is defined with *i, j* = (1, …, *N*):

(1) $$r_{i,j}=\frac{\sum_{k\;=\;1}^{D}\left(s_{i}^{k}-{\bar{s}}_{i})(s_{j}^{k}-{\bar{s}}_{i}\right)}{\sqrt{\sum_{k\;=\;1}^{D}{\left(s_{i}^{k}-{\bar{s}}_{i}\right)}^{2}}\sqrt{\sum_{k\;=\;1}^{D}{\left(s_{j}^{k}-{\bar{s}}_{i}\right)}^{2}}}$$

The numerator is the sum over the product of the voxels intensities $s_{i}^{k}$ and $s_{j}^{k}$ at each voxel position *k* after subtraction of the patch average values, and the denominator is the product of the standard deviations of *s*_*i*_ and *s*_*j*_ gray-level distributions.

Pearson's correlation was chosen to model the effects of atrophy, as it is fast to implement and compute, simple to understand and interpret, and it does not require any scaling or centering of the patches as it is intrinsically normalized. In addition, correlation is a similarity criterion that associates corresponding voxels within patches, therefore taking into account spatial relationships between voxels. To investigate the importance of preserving spatial voxel correspondence when building the multiplex, a preliminary study about similarity metrics had been previously performed (see Supplementary Material), which demonstrated that Pearson's correlation was the optimal choice.

**Figure 4:**
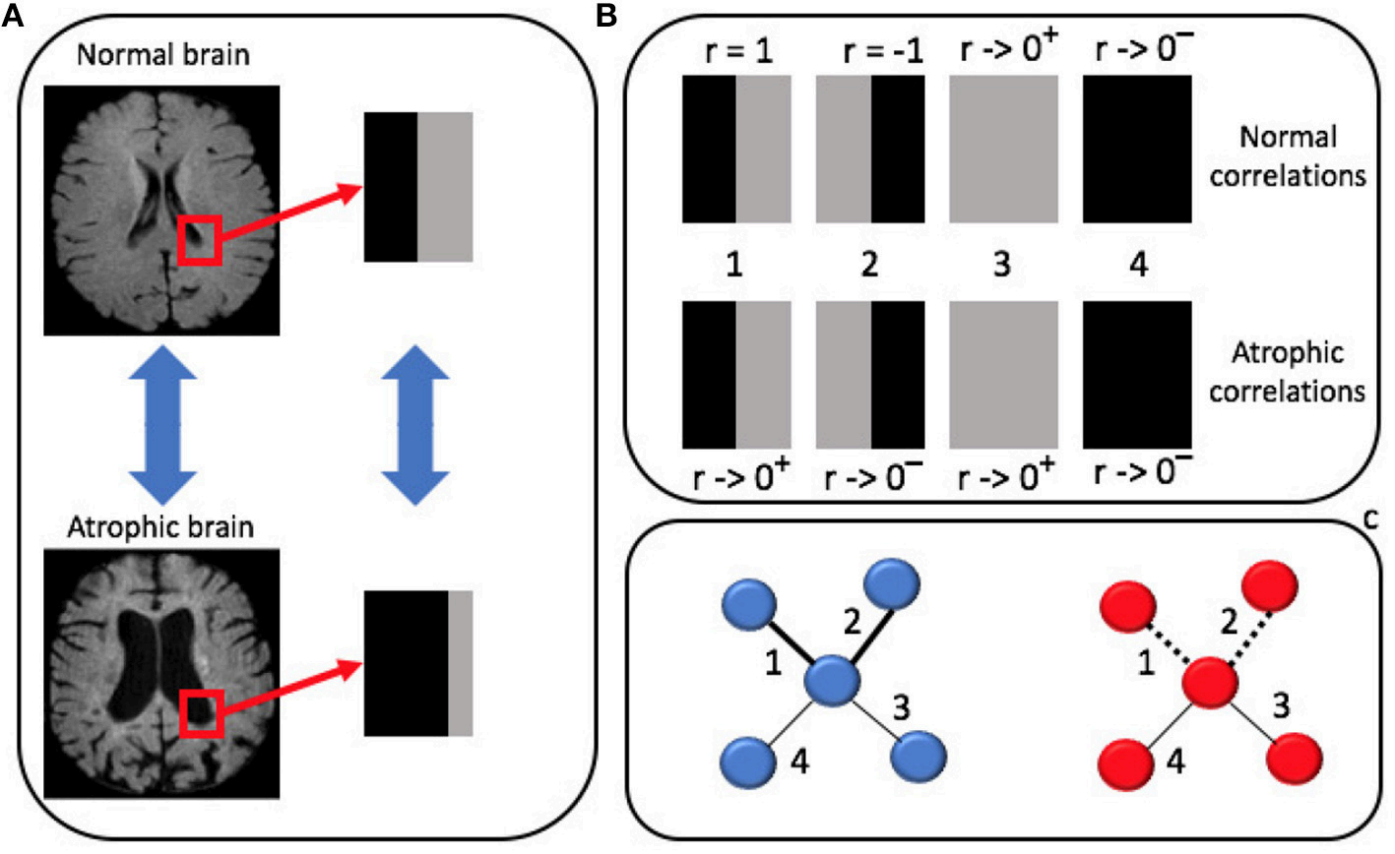
**(A)** Brain morphological changes occur in localized regions and affect the spatial distribution of gray level intensities. For example, atrophy increases the cerebrospinal fluid (CSF) volume at the expenses of gray matter (GM) in panel. **(B)** Pearson's correlation of these two patches is computed against: (1) a patch with a symmetric distribution of GM and CSF; (2) an anti-symmetric patch mimicking left-right inversion; (3) a pure GM patch; (4) a pure CSF patch. **(C)** In atrophic brains (red) connections (1) and (2) disappear (dotted lines) while they remain strong connections in normal brains (blue).

Pearson's correlation admits negative values, thus in principle it could be adopted for a directed weighted network description. In the case discussed here, it is worth noting that negative correlations can be found, for example, between patches in which gray matter and white matter undergo a left-right inversion. As a result, distinguishing positive and negative correlations would have included in the multiplex model a left-right bias. As asymmetry is a common characteristic of atrophy in AD, it was decided to consider undirected networks (see Figure 4).

Network edges can be weighted or unweighted. Unweighted network topology is easier to study and interpret, and has computational advantages. On the one hand, even if in several cases the decision to binarize a weighted network with a suitable threshold could be appropriate, this would seem a forced decision in our case, with the patch similarity being an intrinsically continuous measure. On the other hand, weighted networks can include weak relationships that might be spurious and introduce noise into the graph. Therefore, we decided to threshold the networks by setting to 0 all connections whose absolute correlation was less than moderate (|*r*| < 0.3), in order to exclude noisy interrelationships in the model, and reducing as much as possible the loss of important links. For higher correlations, weights were kept in the model, thus resulting in a weighted undirected network representation for each subject:

(2) $$w_{ij}=\{\begin{array}{ll} 0, & \text{if }\left|r_{ij}\right|\leq \text{ 0.3}\\ r_{ij}, & \text{otherwise} \end{array}$$

An investigation on how the threshold affects the multiplex network ability to detect diseased patterns is reported in the following section 3.1.

In a multiplex it is possible to introduce several topological characteristics that are usually adopted to describe a complex network (Menichetti et al., 2014; Amoroso et al., 2018). In our approach we employed the following indicators: the strength $s_{i}^{\alpha }$ and the inverse participation ratio $Y_{i}^{\alpha }$ of a node *i* in layer α:

(3) $$\begin{array}{lll} s_{i}^{\alpha } & = & \overset{N}{\underset{j=1}{\sum }}w_{ij}^{\alpha } \end{array}$$

(4) $$\begin{array}{lll} Y_{i}^{\alpha } & = & \overset{N}{\underset{j=1}{\sum }}{\left(\frac{w_{ij}^{\alpha }}{s_{i}^{\alpha }}\right)}^{2} \end{array}$$

Strength measurements denote which nodes are more relevant within the network describing a single layer (i.e., a subject) of the multiplex. Inverse participation ratio attains the heterogeneity of the weight distribution within each layer.

Along with these two measurements we also evaluated the conditional means of strength *s*(*k*)^α^ and inverse participation *Y*(*k*)^α^ against the nodes with degree *k*:

(5) $$\begin{array}{lll} s{\left(k\right)}^{\alpha } & = & \frac{1}{N_{k}}\overset{N}{\underset{i=1}{\sum }}s_{i}^{\alpha }\delta \left(k_{i}^{\alpha },k\right) \end{array}$$

(6) $$\begin{array}{lll} Y{\left(k\right)}^{\alpha } & = & \frac{1}{N_{k}}\overset{N}{\underset{i=1}{\sum }}Y_{i}^{\alpha }\delta \left(k_{i}^{\alpha },k\right) \end{array}$$

Summation is extended over the *N*_*k*_ nodes having degree *k*; as summation includes a Kronecker δ function, the only non-null terms, for both strength and inverse participation, are referred to nodes *i* of the layer α whose degree is *k*. These quantities help to understand how weights are distributed within each layer, thus, for example, distinguishing whether, on average, the weights of central nodes and less connected nodes are identically distributed or not. Several studies have already pointed out, especially with group-wise single layer approaches (Tijms et al., 2013a), how these features can describe significant differences among healthy and diseased subjects.

However, it is reasonable to assume that further evidence of significant differences between subjects, can arise from the context information provided by the multiplex framework. Accordingly, this information content was exploited by considering the aggregate adjacency matrix $A^{multi}=a_{ij}^{multi}$ where:

(7) $$\begin{array}{l} a_{ij}^{multi}=\left\{1\;if\;\exists \alpha |w_{ij}^{\alpha }>0\;\wedge \;0\;otherwise\right\} \end{array}$$

The matrix *A*^*multi*^ naturally allowed us to re-introduce the previous measurements within a global perspective. In fact, it was possible to compute for each node an aggregated degree and then use it to weight the previously defined strength and inverse participation. Analogously, we used *A*^*multi*^ to define the aggregate degree for each node and then re-computing the conditional means. In this way we introduced in the description of each node the information produced by the whole multiplex.

In conclusion each network was described by 8*N* features (4*N* single layer and 4*N* multiplex features), resulting in a *M* × 8*N* feature representation which from now on we will call $F_{train}$. It is worthwhile to note that this characterization was independent from the clinical status of the subjects as the multiplex had been built blindly to diagnosis. This base of knowledge was then investigated with supervised machine learning models to extract specific disease effect patterns.

### 2.4. Assessment and validation

The multiplex characterization of the images yielded a simple matrix representation, which could be used to feed machine learning models, and unveil discriminating anatomical patterns.

**Figure 5:**
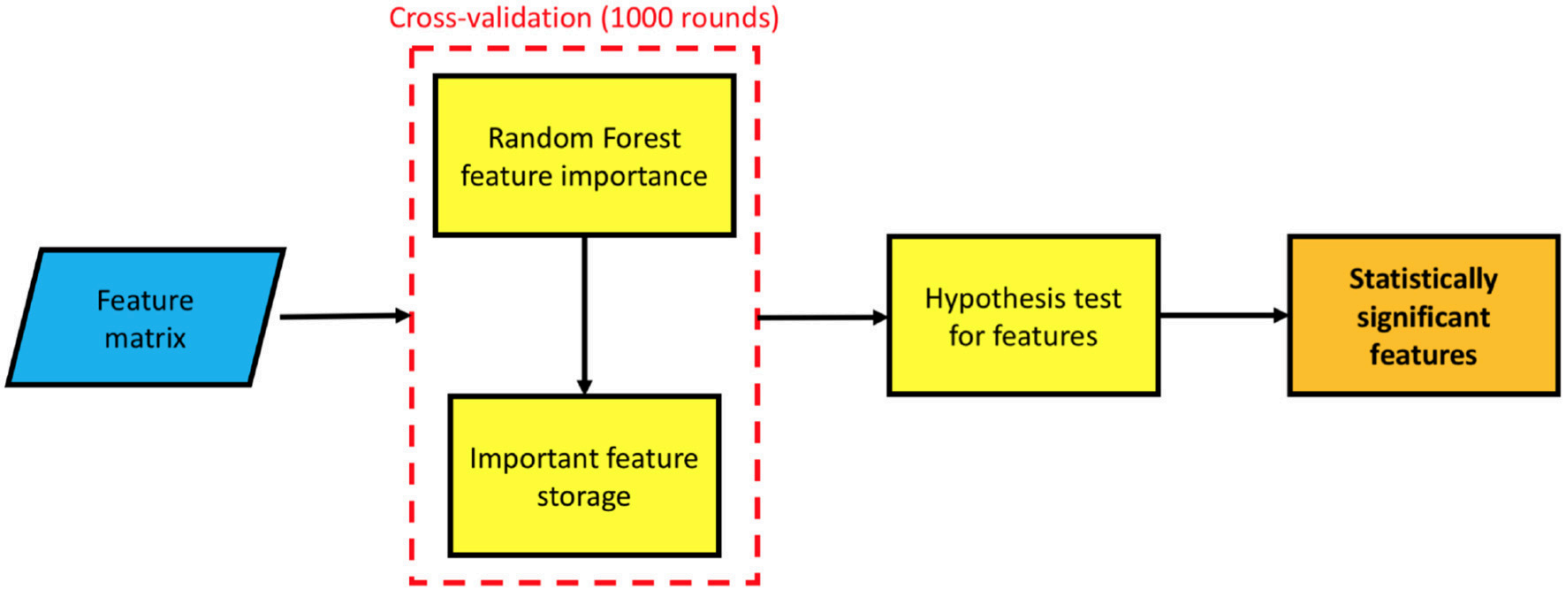
A flowchart of the feature selection methodology: the features, stored in a matrix, are used to train a random forest model, this model provides a feature important estimation; the procedure is cross-validated with a 5-fold for 1, 000 times, at each round taking into account the selected feature. Finally, a statistical test of hypothesis establishes which features have been selected a significant number of times.

The number of features *f*, involved in this approach, could easily reach values ranging from ~10^3^ to ~10^4^ outnumbering the number of the available training samples. Thus, to prevent over-training issues, arising from the curse of dimensionality and assess the multiplex framework, a feature selection was necessary. A flowchart of the whole feature selection method is represented in Figure 5.

A 5-fold cross-validation feature importance selection was performed within a wrapper-based strategy. We randomly divided 1, 000 times $F_{train}$ in a training and a validation test. For each cross-validation round, we built a multiplex model on training subjects, then we computed the important features. In particular, we measured the total decrease in node impurities, in terms of Gini index, from splitting on the variable, averaged over all trees. The selected features were stored for later use and used to train a second random forest classifier which was used to predict the diagnosis of the validation subjects. An evaluation of the informative content of this representation is presented in section 3.2. In both cases random forests were grown with 500 trees, a number large enough for the out-of-bag error to reach the typical training plateau. At each split $\sqrt{f}$ features were randomly sampled.

As previously mentioned, for each cross-validation round different features were selected, thus a quantitative criterion was necessary to determine the most important features, independently from training set. This problem was solved by taking into account the overall occurrence rate of each feature and interpreting it as a success rate. As a consequence a binomial distribution was observed and an experimental *p*-value could be computed to test the randomness hypothesis. We tested it with a *p* < 0.01 to select a more exiguous number of features, then we established which ones had shown a significant probability of occurrence. Once the best features had been selected, we used them to train a new ensemble model on $D_{train}$ and tested it on $D_{test}$ to assess the method robustness and evaluate the informative content carried by multiplex features.

For test subjects, single layer features were straightforwardly computed. Features accounting the whole multiplex structure were in turn computed adding the test subject to the training multiplex but keeping fixed $F_{train}$. The reason for this choice can be justified considering the perturbation induced by the addition of one layer is small.

It is worth noting that features like strength and inverse participation have a direct interpretation, being directly related to a single patch of the brain network whilst conditional means, by definition, are related to several nodes sharing a common degree *k*. For classification purposes this is not an issue, being based on computed features; on the contrary this is relevant in order to provide an anatomical interpretation and a diagnostic value of the features selected.

### 2.5. Anatomical interpretation

Since the identification of the nodes is based on a purely mathematical approach, it seemed important to investigate the relationship between network features and anatomical areas of interest for the disease.

Nodes, whose features were significantly related to AD, were localized on the reference template and the corresponding atlas. We adopted Harvard-Oxford cortical and sub-cortical structural atlases (Desikan et al., 2006). For conditional mean features, which intrinsically encode the information contained in different nodes, we identified nodes significantly related to AD. Next, for each one, we recorded subject by subject the patches having the degree k used to compute that specific conditional mean feature. Then, we computed an occurrence rate taking into account how many times a patch had been used to compute that conditional mean. At this point, patches significantly correlated to AD were identified by interpreting the occurrence as a success rate, and testing the hypothesis of randomness according to a binomial distribution with *p* < 0.01. This methodology allowed us to detect a restricted number of anatomical districts associated to AD, as shown in section 3.3.

## 3. Results

### 3.1. Threshold assessment

**Figure 6:**
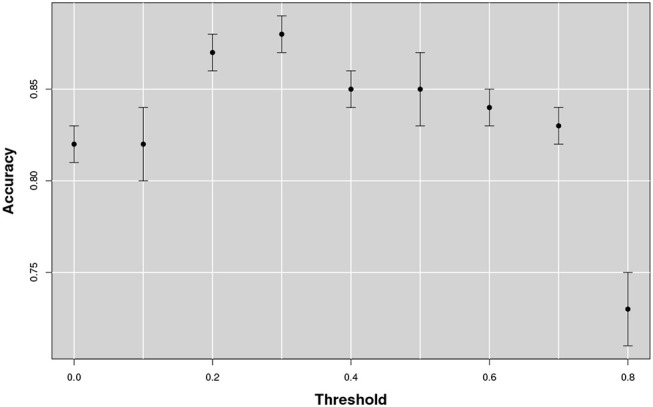
The figure shows the accuracy as a function of the threshold that changes from 0 to 0.8. The best accuracy is obtained in correspondence of a threshold value of 0.3.

Since this approach could in principle heavily depend on the threshold value adopted to discard negligible correlations, the threshold values ranging from 0 to 0.8 were explored with a 0.1 step. Then, for each threshold value a different multiplex was constructed. The patch dimension adopted was 3, 000 mm^3^. The training classification performance was measured in terms of accuracy, see Figure 6.

The classification accuracy reached its maximum value with a 0.3 threshold value and it remained stable over 0.85 for a large range of correlations [0.2, 0.5]. With lower or higher threshold performances showed a significant decrease, especially above the 0.8 threshold; in which case more of the 50% of the networks resulted empty.

### 3.2. Scale selection and informative content

**Figure 7:**
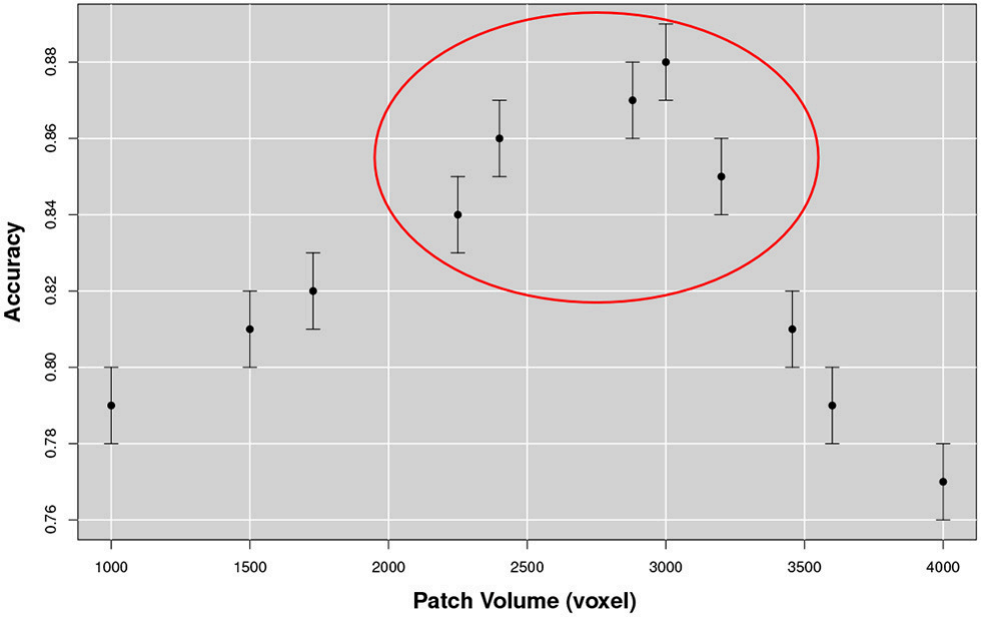
The figure represents the accuracy for the NC-AD classification as a function of the patch size. The existence of a robust plateau, in correspondence of [2, 250, 3, 200] voxels, is highlighted in the circle. These results suggest the existence of an optimal dimensional scale for multiplex describing AD atrophy patterns.

Firstly, we investigated on training the optimal number of nodes *N* to be adopted and, secondly, whether the features thus arising could be used to distinguish NC and AD subjects on the available datasets. This is because the number of nodes *N* of the multiplex, as well as the correlation measure among the different patches, depends on the patch size. As there was no *a priori* reason to choose the patch size, we examined to which extent the size of the patch affected the classification accuracy in discriminating healthy controls and AD subjects from the training data subset (see Figure 7).

From this analysis we found that the optimal size for the patch was of 10 × 15 × 20 mm^3^ equal to an overall volume of 3, 000 mm^3^. Accuracy increased with the patch size until the range [2250, 3200] mm^3^ was reached. At this scale, discarding the patches overlapping the template brain with less than 10% of voxels, 549 patches were obtained for each image. The corresponding accuracy value was on average 0.88 with a 0.01 standard error and a sensitivity and a specificity respectively of 0.90±0.01 and 0.88±0.02. We compared this performance using 180 structural morphological features, obtained by FreeSurfer (6.0 version) (Fischl, 2012), with the same classification strategy, including a first random forest wrapper for feature selection and a second random forest classifier for prediction. In this case classification performance was on average significantly lower 0.83±0.01 confirming the effectiveness of the multiplex characterization.

### 3.3. Anatomical characterization

**Figure 8:**
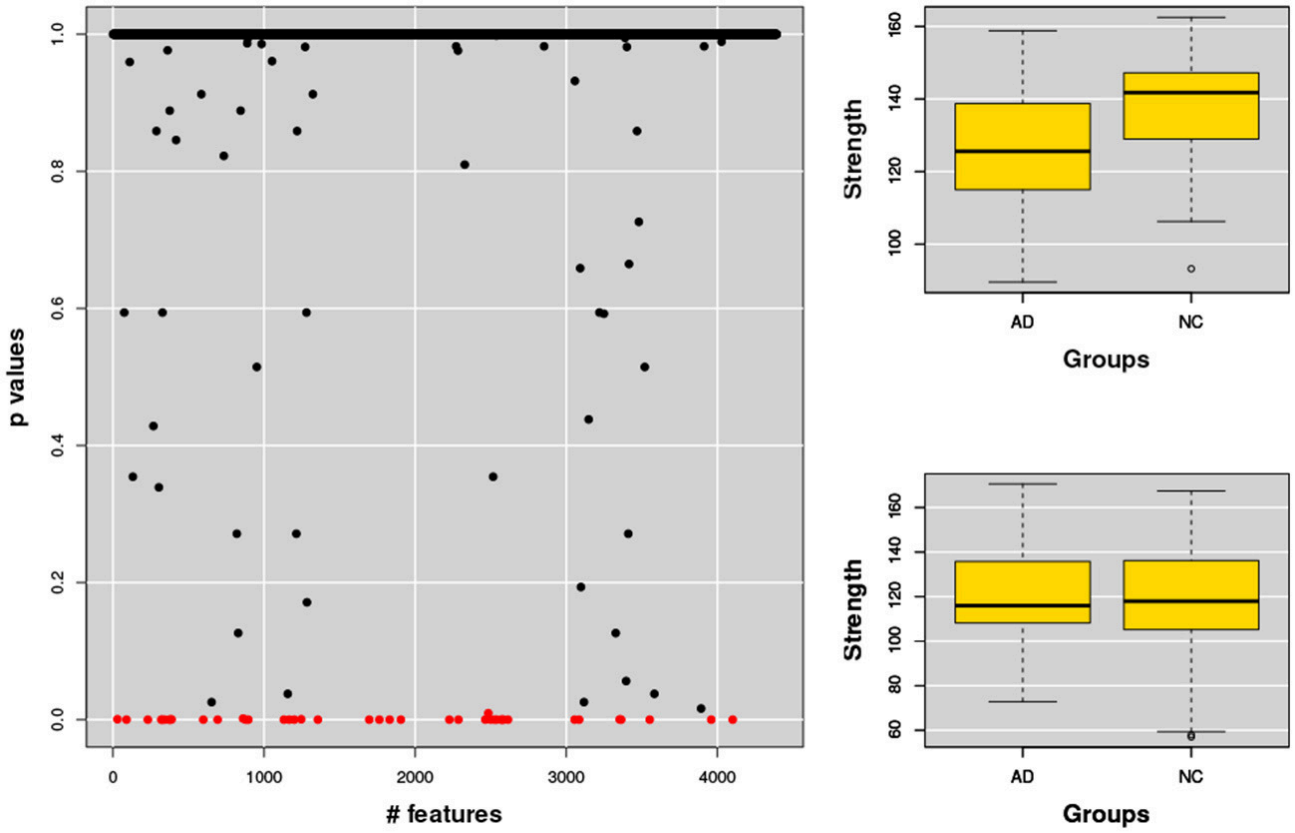
The figure shows **(left)** the *p*-values assigned to each feature, each feature representing a network property, for example the strength of a node. The same analysis was then performed for the related nodes. Typical examples of strength features for nodes significantly correlated **(top)** or not correlated **(bottom)** to AD are also shown **(right)**.

Once the optimal dimension of multiplex network had been fixed we selected the most representative features according to their relative importance. As explained in section 2.4 we selected those features whose contribution to the classification was considerably distant from the null hypothesis of a random behavior, see Figure 8 for a typical example.

**Figure 9:**
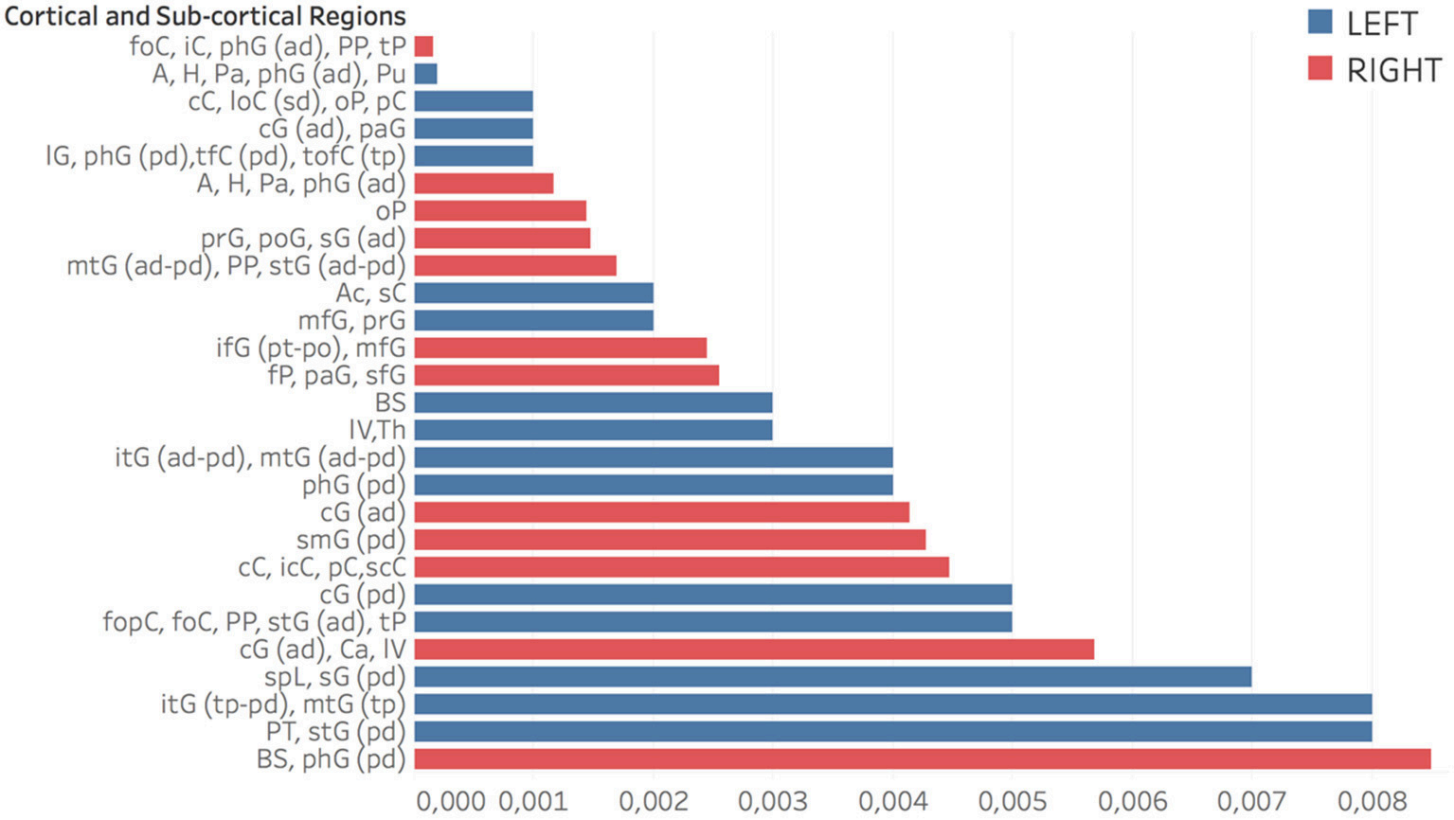
Regions related to AD in order of significance. Accumbens (Ac), Amygdala (A), Brain-Stem (BS), Caudate (Ca), Cingulate Gyrus (cG) anterior division (ad), Cuneal Cortex (cC), Frontal Operculum and Orbital Cortex (fopC) and (foC), Frontal Pole (fP), Hippocampus (H), Inferior Frontal Gyrus (ifG) pars opercularis and pars triangularis (po) and (pt), Inferior Temporal Gyrus (itG) anterior division and temporoccipital part (tp), Insular Cortex (iC), Intracalcarine Cortex (icC), Lateral Occipital Cortex (loC) superior division (sd), Lateral Ventrical (lV), Lingual Gyrus (lG), Middle Frontal and Temporal Gyrus (mfG) and (mtG), Occipital Pole (oP), Pallidum (Pa), Paracingulate and Parahippocampal Gyrus (paG) and (phG), Planum Polare and Temporale (PP) and (PT). Postcentral and Precentral Gyrus (poG) and (prG), Precuneous Coretx (pC), Putamen (Pu), Subcallosal Cortex (sC), Superior Frontal Gyrus (sfG), Superior Parietal Lobule (spL), Superior Temporal Gyrus (stG), Supracalcarine Cortex (scC), Supramarginal Gyrus (sG), Temporal Fusiform and Temporal Occipital Fusiform Cortex (tfC) and (tofC), Temporal Pole (tP), Thalamus (Th). In parentheses: anterior, posterior and superior division (ad,pd,sd) and temporooccipital part (tp).

The whole base of knowledge consisted of 32 significant patches, 18 (~56%) in the left hemisphere and 14 in the right, including 27 different cortical and sub-cortical regions listed in the following Figure 9 in order of significance. As a region can be included in different patches (provided at least one of its voxels belongs to the considered patch), only most significant *p*-value entries are reported.

**Figure 10:**
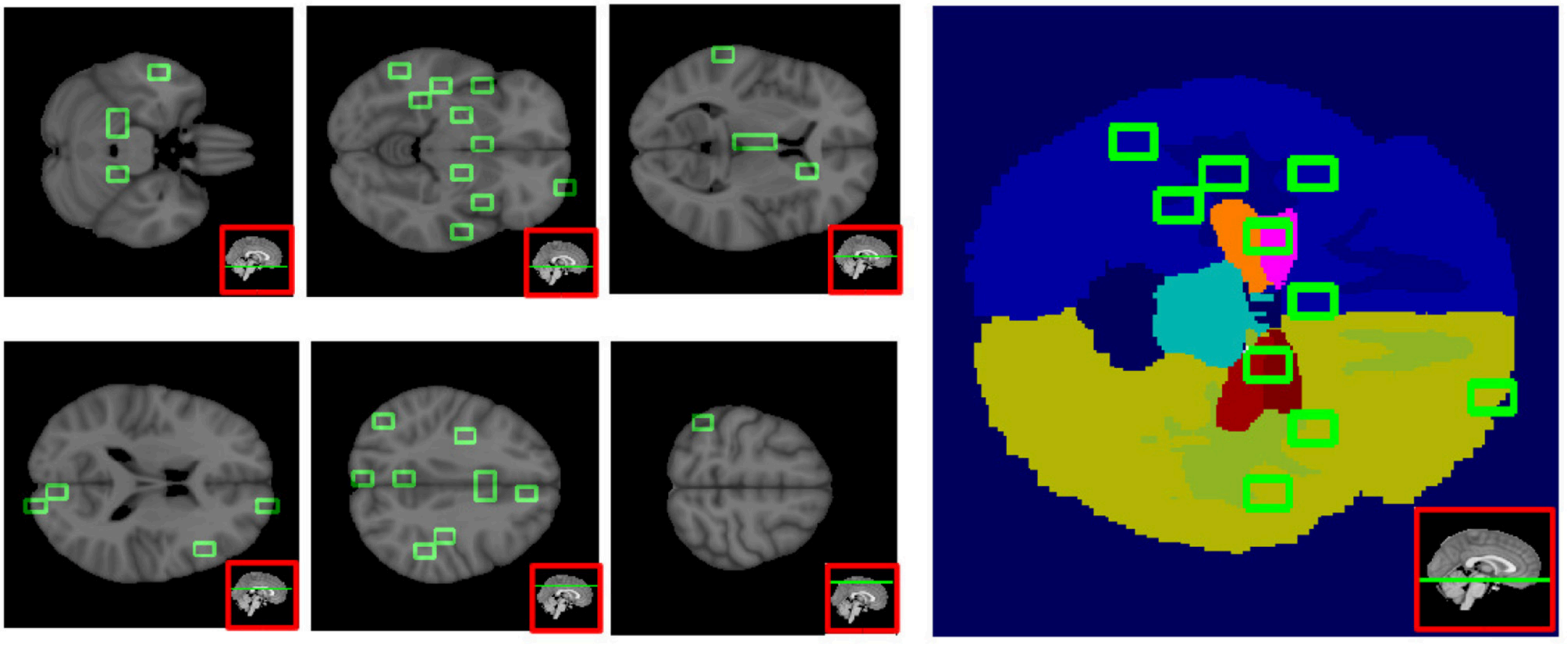
This figure shows six axial planes **(left)** with the significant patches outlined in green (*p* < 0.01), and on the **right**, the Harvard-Oxford Atlas used for the patch anatomical localization.

In Figure 10 some representative brain axial planes are shown, as well as the Harvard-Oxford atlas we used for this assessment. In the left hemisphere, patches corresponding to amygdala, hippocampus, para-hippocampal gyrus, pallidum and putamen showed the strongest association to AD (*p* = 0.0001). For cingulate and para-cingulate giri, pre-cuneus, cuneus, and occipital cortex *p* = 0.001. Other significant patches (*p* = 0.002) were located in middle frontal gyrus and pre-frontal gyrus, nucleus accumbens, brain stem and thalamus.

On the right, *p* = 0.0001 for orbito-frontal cortex, insular cortex, prarahippocampal gyrus, planum polare and planum temporale; *p* = 0.001 for the parahippocampal-amygdalar complex, occipital pole, pre- and post-central gyri, supramarginal gyrus, middle and superior temporal gyri; *p* = 0.002 for inferior, middle and superior frontal gyri, frontal pole, and paracingulate gyrus.

It is interesting to note that frontal lobe involvement was more prominent on the right.

### 3.4. Multiplex networks vs voxel based morphometry

In order to establish if this new approach may offer any advantages over existing widely used methods, we analyzed the same data set with Voxel Based Morphometry (VBM) (Ashburner and Friston, 2000).

**Figure 11:**
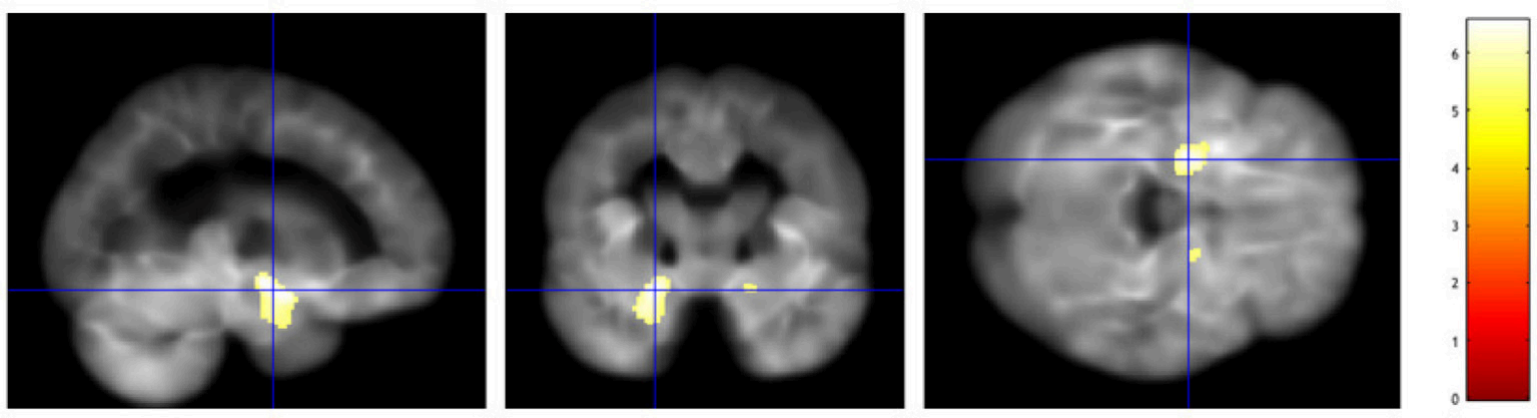
A voxel based morphometry analysis shows bilateral areas of significantly reduced gray matter density in patients with AD, in medial temporal lobe structures, such as hippocampus and amygdala, more prominent on the left as expected.

We followed the standard prescription for VBM with the publicly available SPM 12 suite^1^. Firstly, a segmentation of brain tissues was performed, followed by non-linear normalization with the SPM tool DARTEL to create a study specific template. Secondly, we performed a smoothing with an isotropic Gaussian filter with a full width at half maximum of 8mm. Lastly, a two-sample analysis was performed with a *t* statistics to investigate significant group-wise differences in atrophy between NC and AD on training subjects. Significant voxels, with 5% family-wise correction, are represented below in Figure 11.

The VBM analysis showed significant reduction in gray matter density in bilateral peri-hippocampal regions, more prominent of the left.

### 3.5. Left/Right characterization

Since the VBM analysis confirmed that left-sided changes were more prominent, two dedicated tests were carried out to further explore the lateralization. Firstly, we used the $D_{train}$ to compute the multiplex features, then we selected only those inherent to the left (right) hemisphere and trained the classification models. The feature selection and the cross-validation procedures described in section 2.4 were perfectly replicated as the goal of this test was to quantify the information content of features related to left (right) hemisphere regions. We found that left patches were able to discriminate NC from AD patients with an accuracy of 0.87±0.01while right hemisphere features were able to reach the accuracy value 0.85±0.01. Left hemisphere remained responsible for a greater part of the overall information of the multiplex framework, which was 0.88±0.01.

It must be taken into account that each patch, summarizes a network of interrelationships with other patches independently from its spatial collocation. As an example, the strength of a node denotes the sum of its connections, the fact that a node of the left hemisphere is significantly related to AD does not prevent its strength to be the result of its correlation with the right hemisphere.

As a consequence, a second test was performed. We considered the multiplexes of left and right hemispheres separately. This was done dividing each brain scan in two different images containing the two hemispheres and then using only one half to build the multiplex. Accordingly, the multiplex features computed in this case could be genuinely considered as related to only one hemisphere. Even in this case we performed feature-selection and cross-validation analyses reproducing the whole brain procedure. Classification accuracy for NC-AD when using left multiplex was 0.83±0.01, for right we found 0.81±0.01, thus confirming the greater involvement of the left hemisphere but also signaling a definite deterioration of the information content if compared with the whole brain multiplex.

### 3.6. Robustness and generalization

**Figure 12:**
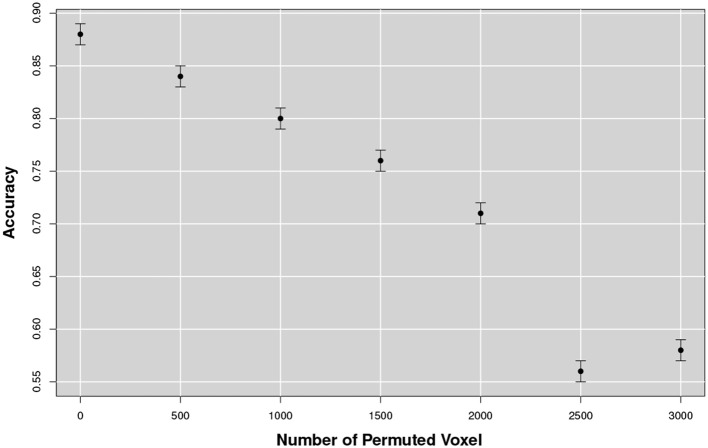
Accuracy varying with the number of permuted voxel within a patch. Classification performance decreased as the number of shuffled voxels was increased. Noticeably, a drastic drop was observed when the shuffle reached values of about 2, 500~3, 000 voxels.

To investigate if classification performance was related to the random permutation of voxels inside a patch, we firstly shuffled a varying number of voxel within each patch, while keeping the patch decomposition stable, thus affecting the Pearson's correlation pairwise measurement. Then we measured the classification accuracy. The training results are presented in Figure 12.

The test was repeated 100times increasing the size of the shuffle by 500voxels at the time. It could be noticed that for small variations, under 1, 000voxels, performance did not suffer a significant deterioration; but with 2, 500 voxel permutation a drastic drop of the performance was observed, a value comparable with the dimensional scale determined in section 3.2.

**Figure 13:**
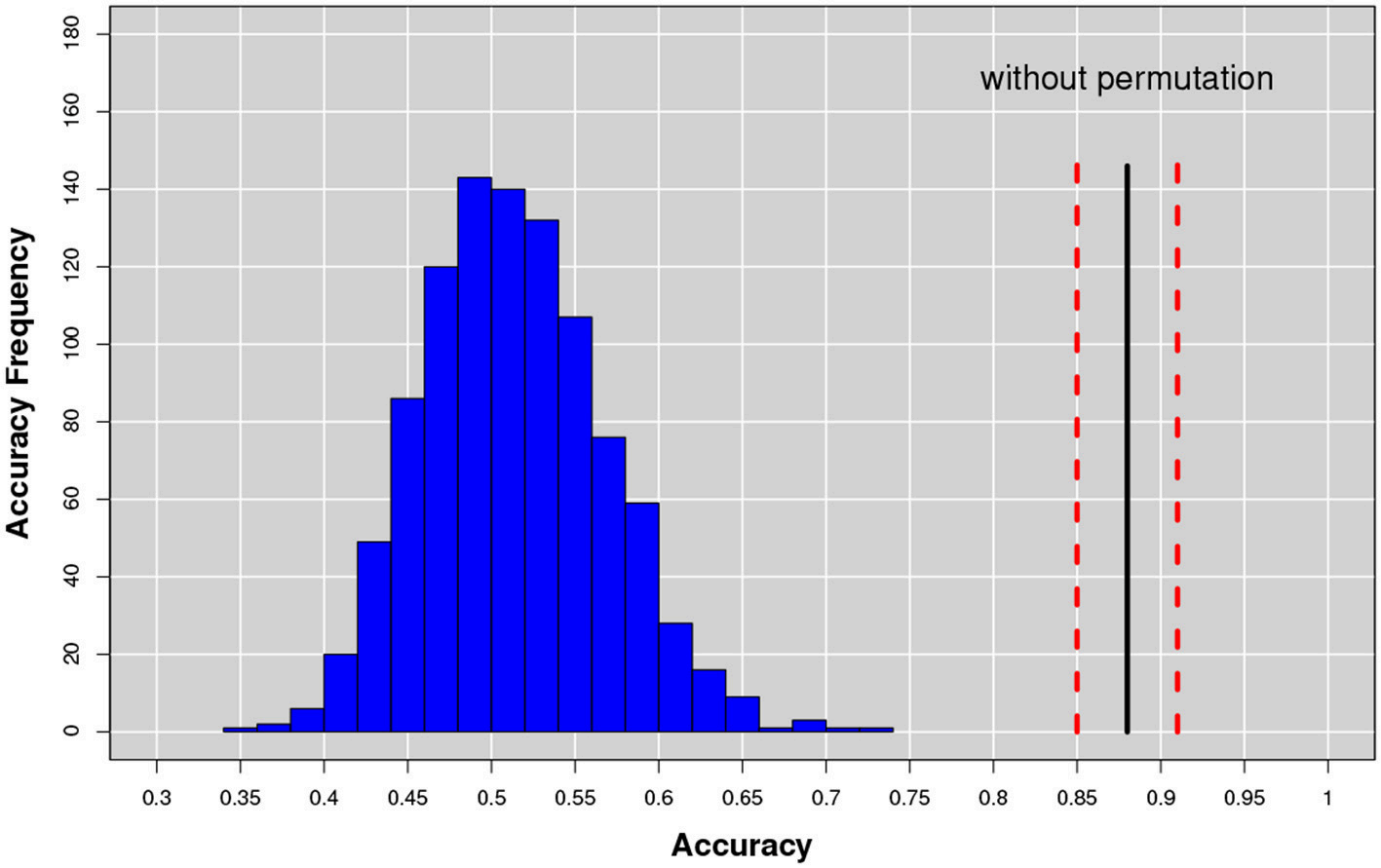
The accuracy distribution for the binary problem NC-AD on the $D_{train}$ with a random permutation of the subject labels. The average value (continuous line) and the relative uncertainty (dotted lines) of best training performances obtained without permutation are also represented for direct comparison.

To further assess the method robustness we also performed a classical non-parametric statistical permutation test. This consisted in the permutation of the clinical labels of each subject belonging to $D_{train}$. We performed 1, 000 random permutation and observed (see Figure 13) a consistent decrease of the classification performance suggesting that the selected features do characterize the disease.

Training set randomization effectively established that the multiplex framework was able to model a significant structure in the $D_{train}$ data between the multiplex features and the clinical label. Moreover, given the normality of the performance distribution obtained by permuting the labels, it was possible to assign a *p*-value to the performance obtained without permutations. The result showed that the multiplex model was able to identify a significant (*p* < 0.001) class structure within the $D_{train}$ data. Otherwise, it would not have been possible to reject the null hypothesis underlying this test, i.e., that labels and features were independent, so that in fact no difference really existed between the classes.

As a further assessment we performed a binary classification on the $D_{test}$ for the NC-AD and NC-cMCI classes. The analysis was repeated using 100 bootstrapped $D_{test}$ sets to provide a measurement of the performance uncertainty. We found in terms of accuracy, respectively 0.86 ± 0.01 and 0.84 ± 0.01. The respective specificity were 0.74 ± 0.01and 0.72 ± 0.01, while sensitivity reached higher values for both cases: 0.96 ± 0.01 and 0.94 ± 0.01. Remarkably, the NC-cMCI classification performance compared well with NC-AD classification confirming the method reliability and its informative content.

The small, but significant, performance deterioration (training accuracy was 0.88 ± 0.01, see section 3.2) could be expected, mainly because even if the test perturbation of the training multiplex was considered small, it should not be completely neglected. The implementation of larger training sets could in principle mitigate this effect. A summary of the classification performances obtained for the different groups are shown in Table 2.

**Table 2:** Summary of the classification performances in terms of accuracy, sensitivity specificity and relative standard errors for the different groups: NC-AD used for the training, NC-AD and NC-cMCI considered for the validation.

**Groups **	**Accuracy **	**Sensitivity **	**Specificity **
$D_{train}\left(NC-AD\right)$	0.88 ± 0.01	0.90 ± 0.01	0.88 ± 0.02
$D_{test}\left(NC-AD\right)$	0.86 ± 0.01	0.96 ± 0.01	0.74 ± 0.01
$D_{test}\left(NC-cMCI\right)$	0.84 ± 0.01	0.94 ± 0.01	0.72 ± 0.01

It is worth noting that these performances were obtained using a subset of 70 features including both single-layer and multiplex features.

## 4. Discussion

The proposed approach aims at modeling brain atrophy in AD through inter-subject multiplex networks whose nodes are represented by brain patches and edges by pairwise Pearson's correlations. Metrics preserving the spatial information as Pearson's correlation and Mutual Information yield accurate results, with the first to be preferred for interpretability and performance consideration. To discard negligible correlations and improve the method sensitivity (as a result of a higher signal to noise ratio) we removed edges with weight below a threshold value of 0.3. Applying this threshold the method appeared robust and the classification performance remained stable over a broad range of correlations ([0.2, 0.5]). Outside this range a performance drop was observed. This is because lower threshold values introduced noisy correlations within the model, thus concealing the effective network information, whilst greater threshold values were too penalizing as informative links were neglected. However, in this study and other similar works (De Vico Fallani et al., 2017), determining the optimal threshold remains an open issue and somehow it limits the robustness of the results.

The method proved to have high sensitivity and high discriminatory power, being therefore suitable both for descriptive and classificatory purposes. As to sensitivity, an optimal volume size for the detection of AD effects, maximizing the informative content of the multiplex, was identified as ranging from 2, 250 to 3, 200 mm^3^. This range can be easily interpreted considering that brain differences may be missed on smaller scales, due for example to misregistration errors; dimensional scales too large, on the contrary, may not capture subtle differences affecting small portions of the brain.

The high sensitivity of the method in the detection of illness related brain changes was demonstrated by the number of regions that were identified as significantly associated with AD. The detected regions comprised hippocampus and para-hippocampal-amygdalar complex, pallidum and putamen, cingulate and paracingulate giri, pre-cuneus, cuneus, and occipital cortex, middle frontal gyrus, pre-central gyrus, accumbens, sub-callosal cortex and brain stem.

While the prominent role in AD pathology of medial temporal lobe structures is widely recognized, the involvement of several other cortical and subcortical areas may be less obvious.

The cingulate cortex is a key component of the default mode network (Buckner et al., 2008), and its early involvement in AD pathology, has been amply demonstrated by functional and structural studies (Minoshima et al., 1997; Yokoi et al., 2018). The same is true for posterior areas, such as cuneus and pre-cuneus, also known to be affected by the illness in early stages (Baron et al., 2001; Bailly et al., 2015). As to the involvement of subcortical gray matter in AD, this has also been recognized, and shown to correlate with cognitive impairment (de Jong et al., 2008). Volume loss of the nucleus accumbens was found to increase the risk of progression from MCI to AD (Yi et al., 2015).

The brain stem is a key area in the early pathophysiology of Parkinson's disease, another common neurodegenerative disorder, and alterations of the brain stem in AD have been shown both *in vivo* (Braun and Van Eldik, 2018), and post-mortem (Simic et al., 2009).

It was striking how VBM on the same data set was able to detect only atrophy of the perihippocampal regions. The method here described seems more sensitive than standard VBM (Good et al., 2002), while studies adopting advanced VBM methodologies have also shown better results (Karas et al., 2003).

The whole base of knowledge consisted of 32regions significant patches, but only 22concerned single-layer measures; the multiplex model thus allowed a consistent increment (+46%) in the detection of significant brain regions.

The results also confirmed asymmetry in the spatial distribution of significant patches, mostly located in the left hemisphere, in keeping with several other studies (Fennema-Notestine et al., 2009; Derflinger et al., 2011; Long et al., 2018). This asymmetry has a direct effect on the informative content.

As to the application of this methodology to disease classification studies, the method is based on the assumption that the introduction of a test subject in the multiplex is not able to significantly perturb the multiplex itself, so that trained models can be easily used for prediction. In fact, on $D_{test}$there is not a great deterioration of the classification performance and the reliability of the framework remains optimal for classification purposes. The framework is robust and accurate, its informative content does not show extreme variations with random shuffling of the voxels inside the patches.

Classification performances are accurate and comparable with recent classification-focused studies (Bron et al., 2015; Moradi et al., 2015; Salvatore et al., 2015; Feng et al., 2018). Even though providing a diagnosis support system is not the main goal of this work, results are encouraging in this sense. Indeed, multiplex model features are able to efficiently capture inter-subject variability underlining disease pattern. An even more refined classification could have been achieved including, as suggested by our previous works, structural features (Amoroso et al., 2014) or longitudinal information (Chincarini et al., 2016).

The method was robust and able to provide a sensitive and informative base of knowledge. This was in particular true when the results were compared with the classification performance using FreeSurfer features. While the present study has been focused of the application of multiplex to disease classification, the method has great versatility and lends itself to a variety of purposes, including the identification of “disease signature” for more anatomically heterogeneous forms of neurodegenerative disorder, such as tauophathies or synucleinopathies, where the model could be enriched with additional clinical or genetic data.

## 5. Conclusion

In this paper we propose a novel approach based on multiplex networks to characterize brain structural variations related to AD. We investigated the information content provided by multiplex networks and showed that they produce an accurate modeling of the disease.

We demonstrated how this framework is able to provide a robust method for AD characterization: (i) it shows the existence of an optimal scale for the description of disease effects of [2, 250, 3, 200] voxels. (ii) Starting from a robust unsupervised brain parcellation, it correctly identifies cerebral region significantly related to AD. It also confirms that AD pathology is more prominent in the left hemisphere. (iii) Multiplex networks are a robust and effective method to describe disease patterns. In fact, after a training phase that gives in cross-validation an accuracy of 0.88±0.01, the multiplex base of knowledge, on the independent dataset $D_{test}$, is able to accurately distinguish between NC and AD subjects with an accuracy of 0.86±0.01and can be suitably employed also for NC and cMCI classification with an accuracy of 0.84±0.01.

The information content provided by multiplex characterization was able to efficiently detect disease patterns. Also the method is very suitable to application to longitudinal studies, ideally in association with functional imaging, to improve our understanding of the different patterns of neurodegeneration in different diseases. The impact of variables such as the degree of atrophy, disease duration, site or scanner type could also be investigated in further studies.

## Ethics statement

All experiments were performed with the informed consent of each participant or caregiver in line with the Code of Ethics of the World Medical Association (Declaration of Helsinki). Local institutional ethics committees approved the study.

## Author contributions

NA and ML conceived and conducted the analyses, SB gave clinical support. All authors NA, RB, SB, ML, TM, AM, and ST analyzed the results and reviewed the manuscript.

### Conflict of interest statement

The authors declare that the research was conducted in the absence of any commercial or financial relationships that could be construed as a potential conflict of interest.

## Footnotes

^1^http://www.fil.ion.ucl.ac.uk/spm/software/spm12/
