# Supplementary material for: Multiplex Networks for Early Diagnosis of Alzheimer's Disease
Source: Front Aging Neurosci. 2018 Nov 14;10:365. doi: 10.3389/fnagi.2018.00365 (PMC6247675; doi:10.3389/fnagi.2018.00365)
Supplement: Supplementary file 1 [file Data_Sheet_1.PDF]

Supplementary Material: Multiplex Networks for early diagnosis of Alzheimer's disease

1 SUPPLEMENTARY DATA

1.1 Similarity Metric Study

To assess the robustness of the proposed methodology, we investigated different similarity measures (Razlighi et al., 2013) to define the presence of an edge between two generic patches s_i and s_j. Using different metrics we were able to build different multiplex models and perform a classification comparison. Classification accuracies and relative standard errors obtained in training are presented in the following Figure S1.

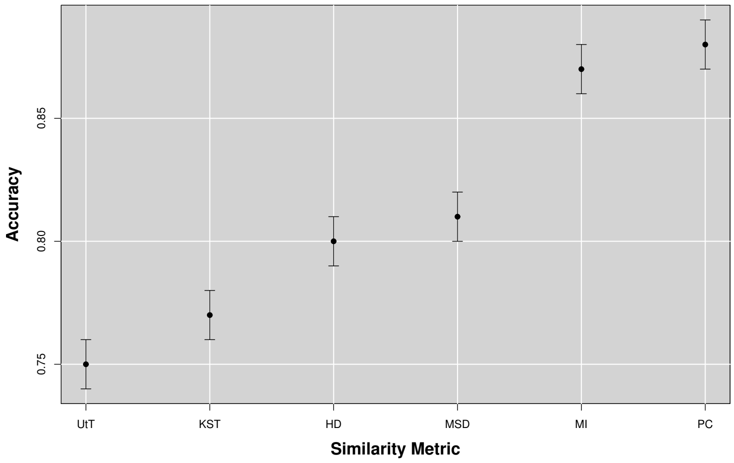

Figure S1. The accuracies with relative standard error for each similarity measurements: Pearson's Correlation (PC), Mutual Information (MI), Mean Square Difference (MSD), Hellinger Distance (HD), Kolmogorov Smirnov non parametric statistic Test (KST), Unpaired t statistic Test (UtT). The best accuracy was obtained using Mutual Information and Pearson's Correlation.

In particular, along with Pearson's correlation (PC), we studied:

- *Mutual Information* (MI):

$$MI_{ij} = H(s_i) + H(s_j) - H(s_i, s_j)$$

  where $H(s_i)$ and $H(s_j)$ are the Shannon entropies related to the patches s_i, s_j and $H(s_i, s_j)$ is their joint Shannon entropy.

- *Mean Square Differences* (MSD):

$$MSD_{ij} = \frac{1}{D}\sum_{k=1}^{D}(s_i^k - s_j^k)^2$$

with s_i^k, s_j^k being the voxel intensity within a patch and D the total number of voxels.

- *Hellinger distance* (HD):

$$HD_{ij} = \frac{1}{D}\sqrt{\sum_{k=1}^{D}(\sqrt{s_i^k} - \sqrt{s_j^k})^2}$$

as usual D is the patch size and s_i^k, s_j^k are the voxel intensities.

- *Kolmogorov Smirnov non parametric statistic test* (KST) quantifying the shape difference between gray level distributions of the patch pairs.
- *Unpaired t statistic test* (UtT) evaluating the difference between means of the patch gray level distribution pairs in terms of standard error.

Best results were obtained by PC and MI which are intrinsically normalized and are able to exploit the spatial correspondence of voxels within a patch. MSD and HD, lacking normalization, suffer a significant performance deterioration. KST and UtT consider respectively only the shape and the average of the gray level distribution of a patch and gave poor results. A complete summary of the metric study, including sensitivity and specificity, is reported in Table S1:

**Similarity Metric**	**Accuracy**	**Sensitivity**	**Specificity**
Pearson's Correlation	$\mathbf{0.88 \pm 0.01}$	$\mathbf{0.90 \pm 0.01}$	$\mathbf{0.88 \pm 0.02}$
Mutual Information	0.87 ± 0.01	$\mathbf{0.90 \pm 0.01}$	0.87 ± 0.02
Mean Square Differences	0.81 ± 0.01	0.84 ± 0.01	0.80 ± 0.02
Hellinger Distances	0.80 ± 0.01	0.83 ± 0.01	0.77 ± 0.02
Kolmogorov Smirnov test	0.77 ± 0.01	0.82 ± 0.01	0.77 ± 0.02
Unpaired t test	0.75 ± 0.01	0.79 ± 0.01	0.73 ± 0.02

Table S1. For each similarity measurement, accuracy sensitivity and specificity with relative standard errors are shown. Best performing metrics are indicated in bold.

REFERENCES

Razlighi, Q. R., Kehtarnavaz, N., and Yousefi, S. (2013). Evaluating similarity measures for brain image registration. *Journal of visual communication and image representation* 24, 977–987
